# Gender Differences in Depression and Sex Hormones among Patients Receiving Long-Term Opioid Treatment for Chronic Noncancer Pain in Taiwan—A Multicenter Cross-Sectional Study

**DOI:** 10.3390/ijerph18157837

**Published:** 2021-07-23

**Authors:** Shung-Tai Ho, Tso-Chou Lin, Chun-Chang Yeh, Kuang-I Cheng, Wei-Zen Sun, Chun-Sung Sung, Yeong-Ray Wen, Yi-Jer Hsieh, Po-Kai Wang, Yen-Chin Liu, Yu-Chuan Tsai

**Affiliations:** 1 Department of Anesthesiology, Kaohsiung Medical University Hospital, Kaohsiung Medical University, Kaohsiung 80756, Taiwan; shungtai0617@gmail.com (S.-T.H.); kuaich@gmail.com (K.-I.C.); 2 National Defense Medical Center, Department of Anesthesiology, Tri-Service General Hospital, Taipei 11490, Taiwan; anes2yeh@gmail.com; 3 Health Science & Wellness Center, Department of Anesthesiology, National Taiwan University Hospital, National Taiwan University, Taipei 10617, Taiwan; wzsun@ntu.edu.tw; 4 Department of Anesthesiology, Taipei Veterans General Hospital, Taipei 11217, Taiwan; sung6119@gmail.com; 5 Department of Anesthesiology, China Medical University Hospital, China Medical University, Taichung 40447, Taiwan; yray.wen@gmail.com; 6 Department of Anesthesiology, Changhua Christian Hospital, Changhua 500209, Taiwan; 67259@cch.org.tw; 7 Department of Anesthesiology, Hualien Tzu Chi Hospital, Buddhist Tzu Chi Medical Foundation, School of Medicine, Tzu Chi University, Hualien 97004, Taiwan; pk8511034@yahoo.com.tw; 8 Department of Anesthesiology, National Cheng Kung University Hospital, National Cheng Kung University, Tainan 70403, Taiwan; anesliu@gmail.com; 9 Center of Pain Management, Department of Anesthesiology E-Da Cancer Hospital, School of Medicine, I-Shou University College of Medicine, Kaohsiung 82445, Taiwan; t3589476@gmail.com

**Keywords:** chronic pain, opioid, gender difference, depression, sex hormone

## Abstract

Background: Long-term use of opioids for chronic noncancer pain is associated with sex hormone disturbances. The interferences with sex hormones, sexual function, and depression were investigated in patients with chronic noncancer pain. Methods: A cross-sectional multicenter survey was conducted on 170 officially registered outpatients receiving long-term opioid treatment in nine medical centers in Taiwan between October 2018 and July 2019. Serum sex hormone levels were examined after the collection of self-administered questionnaires containing the Taiwanese version of the Brief Pain Inventory, depressive status, and sexual function interference. Results: Among 117 (68.8%) questionnaire responses from 170 enrolled outpatients, 38 women and 62 men completed the sex hormone tests, among whom only 23 (23%) had previously received blood hormone tests. Low serum total testosterone levels were detected in 34 (89.5%) women (<30 ng/dL) and 31 (50%) men (<300 ng/dL). Over 60% of women and men reported reduced sexual desire and function despite a nearly 50% reduction in pain intensity and daily function interference over the previous week after opioid treatment. Women generally had higher risks of a depression diagnosis (*p* = 0.034) and severe depressive symptoms (*p* = 0.003) and nonsignificantly lower opioid treatment duration (median 81 vs. 120 months) and morphine milligram equivalent (median 134 vs. 165 mg/day) compared with men. Conclusions: This survey demonstrated the high prevalence of depression diagnosis, low sex hormone levels, and reduced sexual function among Taiwanese patients with chronic noncancer pain receiving prolonged opioid therapy. Regular hypogonadal screenings are recommended for further management.

## 1. Introduction

The drastic increase in opioid prescription and prolonged use over the past two decades has led to an “opioid crisis” in the United States, manifested by prescription opioids involved in 32% of opioid-related overdose deaths in 2018 [1]. Long-term use of opioids contributes to the increased diagnosis and treatment of opioid-induced hypogonadism [2] with clinical presentation of fatigue, depression, sexual dysfunction, and infertility [3]. Meanwhile, reduced gonadal function in menopausal women and older men is associated with sleep disturbances and cognitive decline [4]. Chronic pain, depression, sleep disturbance, sexual dysfunction, and resultant psychosocioeconomic factors all participate in the complexity of unintentional overdose of opioids or suicide [5].

Testosterone and estrogen were traditionally considered male and female sex hormones, respectively. Following the physiological decline of serum levels with advancing age, estrogen treatment exhibits benefits for sexual function in postmenopausal women, [6] and testosterone supplements in men with hypogonadism significantly improve erectile function and libido [7]. A population-based study reported that the prevalence of low testosterone levels was 35% in men receiving long-term opioid treatment, 28% in opioid-unexposed men, [8] and up to 57% among men receiving long-acting opioids [9]. The data for women are insufficient and limited to low testosterone levels (82%), [8] reduced libido (61–100%), and amenorrhea (23–71%) [10]. Despite the abundant literature on hormone treatment for prolonged opioid users, [11] routine hormone screening in patients with chronic noncancer pain (CNCP) is surprisingly uncommon in medical centers (17%) and pain clinics (38%) [2,12].

The National Health Insurance of Taiwan provides comprehensive coverage for the 23 million people residing in Taiwan, including vulnerable patients with CNCP. Long-term use of opioids in patients with CNCP has been strictly regulated in Taiwan since 1996 [13]. Each CNCP outpatient can only obtain prolonged opioid prescriptions from one physician at a medical center or regional hospital but not from physicians in local clinics. The hospital’s narcotic management committee regularly audits each patient’s opioid therapy and psychiatric evaluation and submits the reports to the Taiwan Food and Drug Administration for monitoring [13]. Nevertheless, concerns regarding long-term opioid use in patients in Taiwan with CNCP are emerging in response to the raging opioid crisis in the United States [14].

The history of using questionnaires to interview registered CNCP outpatients in Taiwan extends back to 2001 [15,16,17]. However, few hypogonadism surveys have been conducted in Taiwan, [18] particularly among patients with CNCP. Because of the increase in opioid consumption [19] and the growing population of registered CNCP outpatients in Taiwan, [15,16] this study investigated the long-term use of opioids, examined blood sex hormone levels, and analyzed sex differences in terms of sexual function, daily function, depressive status, and suicidal ideation among Taiwanese patients with CNCP.

## 2. Materials and Methods

### 2.1. Participants

After approval was obtained from the relevant institutional review boards (KMUHIRB-E(II)-20190028, TSGHIRB-2-106-05-162, NTUH-201810037RINC, TVGHIRB-2018-12-010BC, CMUH-108-REC2-029, CCHIRB-181118, HTCH-IRB107-212-B, NCKUHIRB-B-ER-108-008, and EDAHIRB-EMRP-107-118), adult patients receiving long-term opioid treatment for CNCP in nine medical centers from the northern, middle, southern, and eastern regions of Taiwan were enrolled between October 2018 and July 2019. Registered patients with CNCP were identified and briefly interviewed by pain medicine specialists at the outpatient departments. Patients who could not read or write were excluded. After providing written informed consent, participants completed the questionnaire alone or with verbal help from a trained research assistant.

### 2.2. Study Instrument

The Chinese language questionnaire was largely based on previous studies including validation of the Taiwanese version of the Brief Pain Inventory [20] and the Chinese version of the Beck Depression Inventory-II, [21] and our prior surveys [15,16,17]. Pain intensity was evaluated using a numeric scale of 0 (least) to 10 (worst) and the percentage (0–100%) of pain reduction after taking opioids in the past week. The survey also investigated pain-related interference (0 to 10 as least to worst) with daily function, including general activities, mood, ability to walk, normal work activities, relationships with others, sleep, and enjoyment of life, before and after taking opioids in the previous week [20]. We inquired the patients’ depressive score (0–63 in total) by 21 items regarding depressive condition, [21] suicidal ideation, and sexual condition (desire, function, frequency, and satisfaction) over the past week.

Opioid prescriptions were verified by pain medicine specialists at outpatient departments and were then converted to a daily oral morphine milligram equivalent (MME) [22,23,24] using the following conversion factors: intramuscular morphine, 3; codeine, 0.15; oral meperidine, 0.1; intramuscular meperidine, 0.4; oxycodone, 1.5; tramadol, 0.1; fentanyl transdermal (mcg/hour), 2.4; [23] sublingual buprenorphine, 40; transdermal buprenorphine, 2. The 2011 Canadian Guidelines for Safe and Effective Use of Opioids recommend careful reassessment of daily doses approaching 200 mg (defined as the “watchful dose”) [22]. The 2016 CDC guidelines for prescribing opioids for chronic pain revised the high-dose threshold to ≥90 MME per day, at which point justification is required for decisions to titrate the dosage [23].

### 2.3. Blood Hormone Tests

The patients underwent blood hormone tests at the clinical laboratory departments of the original medical centers. Serum levels of sex hormones were examined, including total testosterone, estradiol, luteinizing hormone, and follicle-stimulating hormone. Furthermore, thyroid function hormones, including free T4 and thyroid-stimulating hormone, were tested for opioid-induced endocrinopathy. Testosterone and estradiol levels were assessed as continuous variables. Low serum testosterone levels were defined as <300 ng/dL in men and <30 ng/dL in women, [8] and low serum estradiol levels were defined as ≤20 pg/mL in men, women with their menstrual cycle, [25] and postmenopausal women as the threshold for hormone supplements in Taiwan [26]. A persistently high level of follicle-stimulating hormone (>40 mIU/mL) indicates permanent menopause [26]. Free T4 levels ranged 0.7–1.9 ng/dL were considered normal by the enrolled clinical laboratory departments.

### 2.4. Statistical Analysis

The questionnaire responses were analyzed using SPSS version 22 (IBM Corp., Armonk, NY, USA), with demographic data presented as means ± standard deviation. Pain and interference scores were analyzed between different time and patient groups using paired *t*-tests or one-way ANOVA. The Kruskal–Wallis one-way ANOVA and Mann–Whitney U-test were used to compare MME with sex hormone levels, sexual desire, and depressive scores. Categorical variables were estimated using the chi-squared test or Fisher’s exact test. In all cases, a *p*-Value of <0.05 was considered statistically significant.

## 3. Results

### 3.1. Participant Recruitment

**Figure 1:**
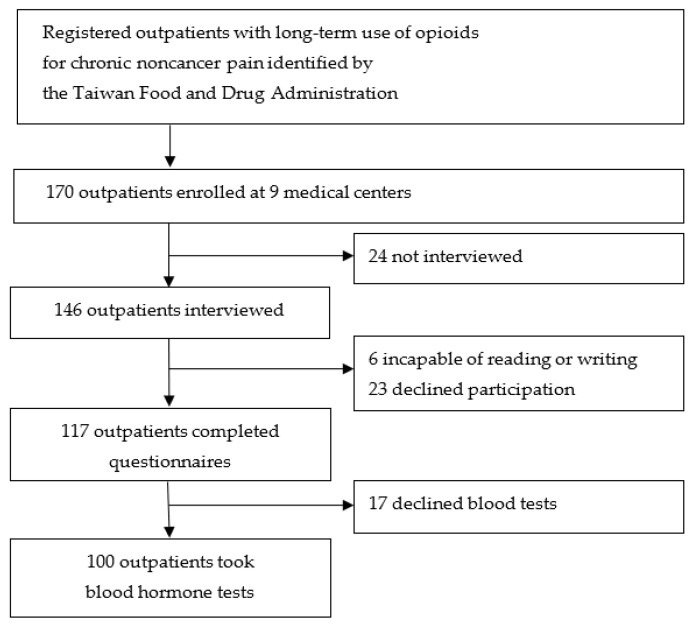
Flow diagram of participant recruitment.

Of 170 CNCP outpatients, 117 (68.8%) completed the questionnaires. Among them, 100 patients (38 women and 62 men) underwent blood hormone tests (Figure 1).

### 3.2. General Characteristics of Participants

Table 1 presents the demographic data, MME, major adjuvant medications, and leading diagnoses for chronic pain in all patients. Women and men were comparable in terms of duration of pain (median: 120 vs. 171 months), duration of opioid therapy (median: 81 vs. 120 months), and daily MME. Approximately 60.5% of women and 72.6% of men had a daily MME ≥ 90 mg. Only 21.1% of women and 24.2% of men underwent blood hormone or endocrine tests before the survey.

### 3.3. Serum Sex Hormone Levels

**Figure 2:**
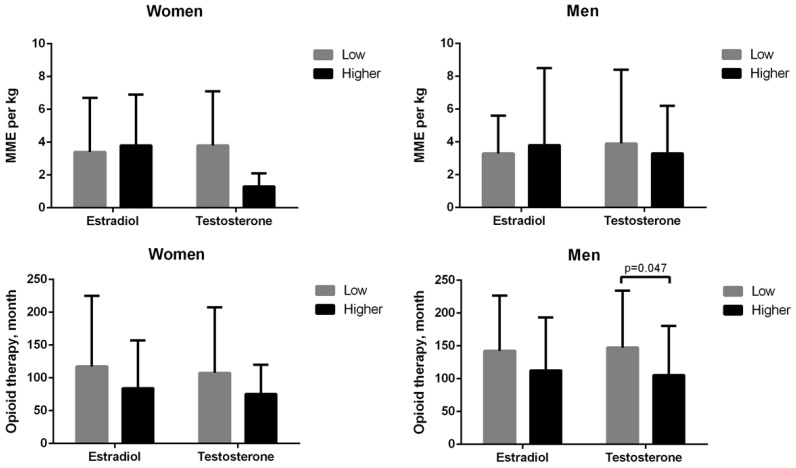
Low serum sex hormone levels were not correlated with a morphine milligram equivalent (MME) per kg (**top**) or duration of chronic opioid therapy (**bottom**) in women or men, except for men with low testosterone levels who had longer durations of opioid therapy. Low serum estradiol levels were defined as ≤20 pg/mL in men and women, and low testosterone levels were defined as <300 ng/dL in men and <30 ng/dL in women.

Table 2 indicates that low serum estradiol levels (≤20 pg/mL) were observed in 1 (11.1%) of 9 women with menstrual cycle, 22 (75.9%) of 29 postmenopausal women, and 29 (46.8%) of 62 men, whereas lower testosterone levels were detected in 34 (89.5%) of 38 women (<30 ng/dL) and 31 (50%) of 62 men (<300 ng/dL). As illustrated in Figure 2, low testosterone levels in men were correlated with a longer duration of opioid therapy (*p* = 0.047). Hypothyroidism was detected in six women and one man without medication. Two women had high free T4 levels despite regular medication.

### 3.4. Depression, Daily Function and Sexual Interferences

Table 3 presents the pain intensity, daily function interference, sexual interference, and depression status during the previous week. Reduced sexual desire was reported by 62 (62%) of 100 patients, and 34 (68%) of 50 sexually active patients reported reduced sexual function, without sex differences. Estradiol levels were lower among women with reduced sexual desire compared with women with increased or unchanged sexual desire (17.1 ± 9.3 vs. 84.1 ± 115.9, *p* = 0.007). Women with estradiol levels below 20 pg/mL were more likely to report reduced sexual desire than women with higher estradiol levels (odds ratio, 5.4; 95% CI, 1.3–22.6; *p* = 0.017). Women had a higher tendency to have a concurrent depression diagnosis (60.5% vs. 38.7%, *p* = 0.034) and severe depressive symptom scores (26.7 ± 15.7 vs. 17.4 ± 12.9, *p* = 0.003) compared with men. Suicidal ideation had been stated as “always/frequently” in 11 (11%) patients, including 5 (13.2%) women and 6 (9.7%) men, without gender differences (*p* = 0.469), in addition to “sometimes” among 12 (31.6%) women and 13 (21.0%) men.

## 4. Discussion

### 4.1. Main Findings

This is the first sex hormone and depression survey among Taiwan registered patients receiving long-term opioid treatment for chronic noncancer pain. Less than one-fourth of the patients had undergone hormone or endocrine tests before this survey. Low serum testosterone levels were detected in 89% of women and 50% of men. Furthermore, over 60% of women and men stated reduced sexual desire, function, and satisfaction. Near half of the patients reported a concurrent depression diagnosis. Women were more likely to have a diagnosis of depression and severe depressive symptoms compared with men. Strong suicide ideation (always/frequently in the past week) in 11% of all patients call our attention to further clinical support and prevention among these registered patients.

### 4.2. Prolonged Opioid Therapy and High Prescribing Rate in Taiwan

Most patients with CNCP in the current study, whether women or men, received prolonged opioid therapy (median 81 vs. 120 months) with a high daily MME (median 134 vs. 165 mg) and prescribed dosage (60% vs. 72%) of MME ≥90 mg/day. All patients received opioid prescriptions from pain medicine specialists. In the United States, pain medicine specialists provided only 8.9% of all opioid prescriptions in 2017, [27] whereas primary care physicians (family medicine, internal medicine, general practice) provided 37.1% and nonphysician prescribers (physician assistant, nurse practitioner) provided 19.2% [27]. In Taiwan, each registered CNCP outpatient can only obtain opioids from one physician in a medical center (tertiary hospital) or regional hospital (secondary hospital), not from any physicians in primary care clinics [28,29]. Furthermore, the pharmaceutical plant of the Taiwan Food and Drug Administration handles the import, export, manufacture, and sale of Schedule 1 and 2 controlled drugs [30]. Patients with CNCP are strictly surveilled. The so-called “pill mills” in the United States are less likely to be developed under Taiwan’s narcotic regulations [13].

### 4.3. Chronic Pain, Depression, Suicide, and Opioid-Related Overdose Deaths

Nearly 50% of the patients in the present study reported a concurrent depression diagnosis, and 30% of all patients were taking antidepressants or benzodiazepines. Pain and sleep deficiency have a bidirectional relationship [31]. Furthermore, the chronic use of opioids (>90 days) is significantly associated with an increased risk of new depression diagnosis [32]. Chronic pain, sleep disturbance, and depression are factors in suicide and unintentional opioid overdose deaths [5]. In 2017, prescription opioids were involved in 36% of opioid-related overdose deaths in the United States, [1] which decreased to 32% in 2018 after immense advocacy for safer prescribing of opioids for chronic pain in the 2016 CDC guidelines [23]. However, stopping opioid treatment was associated with an increased risk of death from overdose or suicide in the United States [33]. The hazard ratio for patients increases with the duration of treatment from 1.67 (≤30 days of treatment duration), 2.80 (31–90 days), 3.95 (91–400 days), to 6.77 (>400 days). Strategies to mitigate the suicide risk in the tapering period are not addressed in the guidelines, despite being a critical concern among patients receiving long-term opioid treatment [33].

### 4.4. Estrogen and Testosterone Levels

Estrogen levels in women fluctuate greatly throughout the menstrual cycle (30–800 pg/mL) until menopause (<20 pg/mL), and in men are relatively constant (<40 pg/mL) [25]. Serum total testosterone levels in men decline with advancing age at a rate of 1–2% per year (i.e., a reduction of 3.2–3.5 ng/dL) from the 30s onward [34]. The prevalence of low total testosterone levels in aging men is, on average, 20% to 30% in the United States [34] and 24% in Taiwan based on the criterion of total testosterone level <300 ng/dL [18]. Following long-term opioid therapy (≥4 weeks), the suppression of the gonadotropin-releasing hormone in the hypothalamic–pituitary–gonadal axis causes a luteinizing hormone, a follicle-stimulating hormone, estradiol, and testosterone deficiencies [3]. Prolonged opioid exposure is associated with increased rates of screening, diagnosis, and treatment for hypogonadism [2,8]. The 2011–2012 National Health and Nutrition Examination in the United States revealed that the incidence of low testosterone levels (<300 ng/dL) was higher among opioid-exposed men than opioid-unexposed men (35.1% vs. 28.3%) [8]. Similarly, opioid-exposed women had a higher incidence rate of testosterone level <30 ng/dL (82.6% vs. 77.0%) as compared with opioid-unexposed women [8]. A meta-analysis revealed that the prevalence of opioid-induced hypogonadism defined by sex hormone levels was 63% (95% CI, 55–70%) among 3250 patients [35]. Most of the results (99.5%) were from testosterone tests in male patients. Only two studies were conducted on women and reported no changes in serum estradiol levels following long-term opioid treatment [35]. In the current study, Taiwanese patients with CNCP exhibited a higher prevalence of low serum estradiol and testosterone levels (61% and 89% in women, and 47% and 50% in men, respectively). Coupling biochemical findings of hormone deficiency with hypogonadal symptoms in patients with CNCP appears to be the best strategy for refining the diagnosis of hypogonadism and potential hormone therapies for older men and women [34].

### 4.5. Sexual Dysfunction

In addition to chronic pain, patients with opioid-induced hypogonadism also present symptoms of depression, muscle wasting, and sexual dysfunction [3]. The only evidence-based indication for testosterone therapy in women is hypoactive sexual desire disorder, which involves a full clinical assessment and cannot be determined according to blood testosterone level alone [36]. In the present study, less than one-quarter of Taiwanese patients with CNCP had undergone endocrine or hormone tests before this survey, and over 60% of women and men reported reduced sexual desire and satisfaction. Based on the high prevalence of reduced sexual desire and function, routine screening of sex hormone levels and sexual symptoms should be indicated for chronic pain management in Taiwan.

### 4.6. Limitations

This uncontrolled and nonblinded cross-sectional study has some limitations that must be addressed. First, selection bias may exist because the participants all received extremely prolonged opioid therapy treatment (median of 81 months in women and 120 months in men). Patients may be more compliant with opioid therapy, which would lead to an overestimation of the effectiveness of opioids and the prevalence of opioid-induced hormone disturbance. Nonopioid medications should be initially prescribed for pain management. Patients with inadequate pain-relief efficacy or shorter duration of opioid therapy are encouraged to seek interventional procedures instead of a high dose of opioids. Second, a limited sample of 100 registered patients from the Taiwan Food and Drug Administration was analyzed. We selected the leading nine medical centers across Taiwan to represent the population of patients with CNCP and investigate the prevalence of opioid-induced hypogonadism. Our findings suggest regular screening of sex hormones among registered CNCP patients in Taiwan under the coverage of Taiwan’s National Health Insurance program. Third, the purpose of this study was to investigate the prevalence of opioid-induced hypogonadism rather than the opioid epidemic and prescription-related mortality among patients in Taiwan with CNCP. Further follow-ups of these participants would provide data on the chronic opioid therapy-related rates of dropout, suicide, or unintentional opioid overdose deaths.

## 5. Conclusions

The findings from this study have implications for clinicians. Long-term opioid therapy provided acceptable pain reduction and improved daily functioning among Taiwanese CNCP outpatients. However, depression diagnosis and low sex hormone levels were detected in over 60% of women. Furthermore, over 60% of women and men reported reduced sexual desire and function. Regular hypogonadal screening of sex hormones and sexual symptoms is recommended for the registered patients with CNCP in Taiwan.

## Figures and Tables

**Table 1:** General data (*n* = 100).

	Women (*n* = 38)		Men (*n* = 62)		*p*-Value	Odds Ratio
	Mean ± SD (Range)	Median	Mean ± SD (Range)	Median		
Age, year	55.4 ± 13.6 (25–94)	57	50.3 ± 9.2 (37–78)	48	0.026 ^a^	0.96
Height, cm	156.8 ± 8.9 (130–168)	158.5	168.5 ± 7.5 (150–185)	168.5	<0.001 ^a^	1.23
Weight, kg	57.9 ± 15.4 (30–97)	56	67.2 ± 15.0 (41–102)	64	0.004 ^a^	1.04
Body mass index	23.4 ± 5.2 (15.6–34.4)	23.4	23.7 ± 5.0 (14.5–38.9)	23.7	0.797 ^a^	
Pain duration, month	140.5 ± 108.4 (12–485)	120	172.0 ± 96.0 (24–480)	171	0.134 ^a^	
Opioid therapy, month	104.3 ± 95.7 (6–384)	81	126.5 ± 83.0 (6–335)	120	0.223 ^a^	
MME, oral, mg/day	194.2 ± 172.1 (15–604)	134	227.6 ± 230.0 (3–1350)	165	0.440 ^a^	
MME per kg, mg/day/kg	3.5 ± 3.2 (0.4–12)	2.5	3.6 ± 3.8 (0.0–24.6)	2.5	0.945 ^a^	
MME ≥ 200 mg/day, *n* = 40	14 (36.8%)		26 (41.9%)		0.614 ^b^	
MME ≥ 90 mg/day, *n* = 68	23 (60.5%)		45 (72.6%)		0.210 ^b^	
Concomitant use of adjuvants, *n* (%)						
Gabapentin or pregabalin	15 (39.5%)		24 (38.7%)		0.939 ^b^	
Tricyclic antidepressants	5 (13.2%)		12 (19.4%)		0.423 ^b^	
Serotonin reuptake inhibitor antidepressants	5 (13.2%)		7 (11.3%)		0.762 ^c^	
Benzodiazepine	9 (23.7%)		20 (32.3%)		0.359 ^b^	
Top 3 diagnoses of pain source, *n* (%)						
Failed back surgery syndrome, *n* = 20	8 (21.1%)		12 (19.4%)		0.837 ^b^	
Chronic pancreatitis, *n* = 15	1 (2.6%)		14 (22.6%)		0.007 ^b^	10.79
Spinal cord injury, *n* = 14	5 (13.2%)		9 (14.5%)		0.849 ^b^	
Blood hormone tests ever received, *n* = 23	8 (21.1%)		15 (24.2%)		0.717 ^b^	

Data are presented as the mean ± standard deviation (SD) (range) or a number (%). MME, morphine milligram equivalent; ^a^. *p*-Values were estimated using Student’s *t*-tests; ^b^. *p*-Values were estimated using a chi-squared test; ^c^. *p*-Values were estimated using Fisher’s exact tests.

**Table 2:** Serum sex hormone levels (*n* = 100).

	Women (*n* = 38)		Men (*n* = 62)		*p*-Value
	Mean ± SD (Range)	Median	Mean ± SD (Range)	Median	
Follicle-stimulating hormone level, mIU/mL					
With menstruation, *n* = 9	8.2 ± 3.6 (3.9–15.6)	7.4	-	-	-
*Clinical menopause, *n* = 29	46.4 ± 28.5 (0.7–117.3)	45.2	-	-	-
Serum estradiol level, pg/ml	41.8 ± 76.5 (5–424)	20	25.7 ± 11.6 (10–52)	21	-
>20 pg/mL, *n*	15 (39.5%)		33 (53.2%)		-
Age < 50, W/M = 8/18	122.4 ± 137.5 (27–424)	46.9	35.0 ± 11.5 (21–52)	34.5	-
Age ≥ 50, W/M = 7/15	42.9 ± 46.1 (24–147)	24.7	30.9 ± 10.0 (21–52)	27.9	-
≤20 pg/mL, *n*	23 (60.5%)		29 (46.8%)		0.182 ^a^
With menstruation, *n* = 1 in 9	1 (11.1%)		-	-	-
Clinical menopause, *n* = 22 in 29	22 (75.9%)		-	-	-
Serum testosterone level, ng/dL	15.2 ± 9.5 (3–40)	13	347.6 ± 273.8 (50–1416)	297	
Age 20–39, W/M = 4/6	14.4 ± 3.6 (10–18)	15	334.3 ± 99.1 (179–474)	339	
Age 40–64, W/M = 29/51	15.2 ± 10.0 (3–40)	10	360.5 ± 296.5 (50–1416)	289	0.527 ^b^
Age 65–79, W/M = 3/5	12.3 ± 8.9 (3–21)	13	232.9 ± 114.6 (86–383)	214	
Age ≥ 80, W/M = 2/0	10, 31		-	-	-
Women < 30 ng/dL, *n*	34 (89.5%)				-
Men < 300 ng/dL, *n*			31 (50.0%)		-

Data are presented as the mean ± standard deviation (SD) (range) or a number (%); ^a^. *p*-Values were estimated using a chi-squared test; ^b^. *p*-Values were estimated using the Kruskal–Wallis test for men’s testosterone levels for three age groups. * Five postmenopausal women with low FSH levels (0.7, 1.5, 2.2, 10.8, and 12.7 mIU/mL).

**Table 3:** Pain intensity reduction, daily function interference, sexual interference, and depressive status over the previous week (*n* = 100).

	Women (*n* = 38)		Men (*n* = 62)		*p*-Value	Odds Ratio
	Mean ± SD (Range)	Median	Mean ± SD (Range)	Median		
Pain intensity reduction after taking opioids, %	43.2 ± 19.6 (0–90)	45	49.5 ± 18.4 (10–100)	50	0.105 ^a^	
Daily function interference, in average						
Before taking opioids, 0–10	7.9 ± 2.1 (3.6–10)	8.4	8.0 ± 2.0 (0.9–10)	8.4	0.696 ^a^	
After taking opioids, 0–10	5.0 ± 2.5 (0–9.6)	4.8	4.7 ± 2.6 (0–10)	4.7	0.610 ^a^	
Interference reduction after taking opioids, %	35.9 ± 26.2 (−18.0–100)	33.5	41.6 ± 29.5 (–33.8–100)	39.5	0.327 ^a^	
Sexual desire, *n* = 100						
Increased or unchanged, *n* = 38	14 (36.8%)		24 (38.7%)			
Decreased, *n* = 62	24 (63.2%)		38 (61.3%)		0.852 ^b^	
Sexual function in those sexually active, *n* = 50	18 (47.4%)		32 (51.6%)		0.680 ^b^	
Increased or unchanged, n = 16	6 (33.3%)		10 (31.2%)			
Decreased, *n* = 34	12 (66.7%)		22 (68.8%)		0.880 ^b^	
Depression diagnosis by now, *n* = 47	23 (60.5%)		24 (38.7%)		0.034 ^b^	0.41
Depression diagnosis before chronic pain, *n* = 30	18 (47.4%)		12 (19.4%)		0.003 ^b^	0.27
Beck Depression Inventory score, 0–63^#^	26.7 ± 15.7 (0–55)	28.5	17.4 ± 12.9 (0–63)	16	0.003 ^c^	0.96
With decreased sexual desire, W/M = 24/38	30.3 ± 16.7 (1–55)	31.5	18.1 ± 14.2 (0–63)	15.5	0.003 ^c^	0.95
With decreased sexual function, W/M = 12/22	26.3 ± 14.2 (6–46)	27.5	15.2 ± 7.8 (1–37)	14.5	0.028 ^c^	0.91
Suicidal ideation, *n* (%)						
Always / Frequently	5 (13.2%)		6 (9.7%)		0.469 ^d^	
Sometimes	12 (31.6%)		13 (21.0%)			
Seldom	5 (13.2%)		14 (22.6%)			
Never	16 (42.1%)		29 (46.8%)			

Data are presented as the mean ± standard deviation (SD) (range) or a number (%). Pain intensity, 0 (no pain) to 10 (worst pain imaginable); ^a^. *p*-Values were estimated using Student’s *t*-tests; ^b^. *p*-Values were estimated using a chi-squared test; ^c^. *p*-Values were estimated using the Mann–Whitney U-tests; d. *p*-Values were estimated using Fisher’s exact tests.

## Data Availability

The data presented in this study are available on request from the corresponding author.

## References

[1] Wilson N., Kariisa M., Seth P., Smith H., Davis N.L. (2020). Drug and Opioid-Involved Overdose Deaths—United States, 2017–2018. MMWR Morb. Mortal. Wkly. Rep..

[2] Baillargeon J., Raji M.A., Urban R.J., Lopez D.S., Williams S.B., Westra J.R., Kuo Y.-F. (2019). Opioid-Induced Hypogonadism in the United States. Mayo Clin. Proc. Innov. Qual. Outcomes.

[3] Antony T., Alzaharani S.Y., El-Ghaiesh S.H. (2019). Opioid-induced hypogonadism: Pathophysiology, clinical and therapeutics review. Clin. Exp. Pharmacol. Physiol..

[4] Hajali V., Andersen M.L., Negah S.S., Sheibani V. (2019). Sex differences in sleep and sleep loss-induced cognitive deficits: The influence of gonadal hormones. Horm. Behav..

[5] Bohnert A.S.B., Ilgen M.A. (2019). Understanding Links among Opioid Use, Overdose, and Suicide. N. Engl. J. Med..

[6] Nastri C.O., Lara L.A., Ferriani R.A., Rosa E.S.A.C., Figueiredo J.B., Martins W.P. (2013). Hormone therapy for sexual function in perimenopausal and postmenopausal women. Cochrane Database Syst. Rev..

[7] Corona G., Rastrelli G., Morgentaler A., Sforza A., Mannucci E., Maggi M. (2017). Meta-analysis of Results of Testosterone Therapy on Sexual Function Based on International Index of Erectile Function Scores. Eur. Urol..

[8] Cepeda M.S., Zhu V., Vorsanger G., Eichenbaum G. (2015). Effect of Opioids on Testosterone Levels: Cross-Sectional Study using NHANES. Pain Med..

[9] Rubinstein A., Carpenter D.M. (2014). Elucidating Risk Factors for Androgen Deficiency Associated with Daily Opioid Use. Am. J. Med..

[10] Wersocki E., Bedson J., Chen Y., LeResche L., Dunn K.M. (2016). Comprehensive systematic review of long-term opioids in women with chronic noncancer pain and associated reproductive dysfunction (hypothalamic–pituitary–gonadal axis disruption). Pain.

[11] Aminilari M., Manjoo P., Craigie S., Couban R., Wang L., Busse J.W. (2018). Hormone Replacement Therapy and Opioid Tapering for Opioid-Induced Hypogonadism Among Patients with Chronic Noncancer Pain: A Systematic Review. Pain Med..

[12] Hochberg U., Ojeda A., Brill S., Perez J. (2018). An Internet-Based Survey to Assess Clinicians’ Knowledge and Attitudes Towards Opioid-Induced Hypogonadism. Pain Pr..

[13] Taiwan Food and Drug Administration Physician Guidelines on Clinical Use of Narcotics in Chronic Noncancer Pain. https://www.fda.gov.tw/tc/lawContent.aspx?cid=183&id=3086.

[14] Taiwan Food and Drug Administration 2019 International Conference on Narcotics: Safe Use and Management. https://www.fda.gov.tw/ENG/newsContent.aspx?id=25571.

[15] Lin T.-C., Hsu C.-H., Lu C.-C., Tsai Y.-C., Ho S.-T. (2010). Chronic opioid therapy in patients with chronic noncancer pain in Taiwan. J. Anesthesia.

[16] Lin T.-C., Ger L.-P., Pergolizzi J.V., Raffa R.B., Wang J.-O., Ho S.-T. (2017). Long-term use of opioids in 210 officially registered patients with chronic noncancer pain in Taiwan: A cross-sectional study. J. Formos. Med Assoc..

[17] Lin T.-C., Ho S.-T., Ger L.-P., Liou H.-H., Hwang S.-L. (2018). Gender difference in long-term use of opioids among Taiwan officially registered patients with chronic noncancer pain. Medicine.

[18] Liu C.-C., Wu W.-J., Lee Y.-C., Wang C.-J., Ke H.-L., Li W.-M., Hsiao H.-L., Yeh H.-C., Li C.-C., Chou Y.-H. (2009). The Prevalence of and Risk Factors for Androgen Deficiency in Aging Taiwanese Men. J. Sex. Med..

[19] Pan H.-H., Ho S.-T., Lu C.-C., Wang J.-O., Lin T.-C., Wang K.-Y. (2013). Trends in the Consumption of Opioid Analgesics in Taiwan From 2002 to 2007: A Population-Based Study. J. Pain Symptom Manag..

[20] Ger L.-P., Ho S.-T., Sun W.-Z., Wang M.-S., Cleeland C.S. (1999). Validation of the Brief Pain Inventory in a Taiwanese Population. J. Pain Symptom Manag..

[21] Beck A.T., Steer R.A., Ball R., Ranieri W.F. (1996). Comparison of Beck Depression Inventories-IA and-II in Psychiatric Outpatients. J. Pers. Assess..

[22] Kahan M., Mailis-Gagnon A., Wilson L., Srivastava A. (2011). Canadian guideline for safe and effective use of opioids for chronic noncancer pain: Clinical summary for family physicians. Part 1: General population. Can. Fam. Physician.

[23] Dowell D., Haegerich T.M., Chou R. (2016). CDC Guideline for Prescribing Opioids for Chronic Pain—United States, 2016. JAMA.

[24] Opioid Dose Equivalence Faculty of Pain Medicine, ANZCA—March 2019. https://www.anzca.edu.au/getattachment/6892fb13-47fc-446b-a7a2-11cdfe1c9902/PM01-(Appendix-2)-Opioid-Dose-Equivalence-Calculation-of-Oral-Morphine-Equivalent-Daily-Dose-(oMEDD).aspx.

[25] Stanczyk F.Z., Clarke N.J. (2014). Measurement of Estradiol—Challenges Ahead. J. Clin. Endocrinol. Metab..

[26] Chang C., Wu P.H., Chang C.H., Tsai T.J., Hsu L.L., Lee M.J. (1999). Hormone levels and menopausal status in middle aged women: A cross-sectional study (in Chinese). Chin. J. Public Health.

[27] Guy G.P., Zhang K. (2018). Opioid Prescribing by Specialty and Volume in the U.S. Am. J. Prev. Med..

[28] Lin T.-C., Ger L.-P., Pergolizzi J.V., Raffa R.B., Wang J.-O., Ho S.-T. (2016). Knowledge, Attitude and Practice Survey of Prescribing Opioids for Chronic Noncancer Pain in Taiwan—Comparison of Pain and Non-Pain Physicians. Pain Med..

[29] Hsu Y.-C., Lin S.-L., Sung C.-S., Ger L.-P., Liou H.-H., Lin T.-C., Ho S.-T. (2020). Knowledge, attitude, and barriers regarding prescribing long-term opioids among Taiwan physicians treating officially registered patients with chronic noncancer pain. J. Chin. Med. Assoc..

[30] Taiwan Food and Drug Administration Controlled Drugs Act. https://www.fda.gov.tw/ENG/lawContent.aspx?cid=5061&id=603.

[31] Haack M., Simpson N., Sethna N., Kaur S., Mullington J. (2019). Sleep deficiency and chronic pain: Potential underlying mechanisms and clinical implications. Neuropsychopharmacology.

[32] Scherrer J.F., Salas J., Schneider D., Bucholz K.K., Sullivan M.D., Copeland L., Ahmedani B.K., Burroughs T., Lustman P.J. (2017). Characteristics of new depression diagnoses in patients with and without prior chronic opioid use. J. Affect. Disord..

[33] Oliva E.M., Bowe T., Manhapra A., Kertesz S., Hah J.M., Henderson P., Robinson A., Paik M., Sandbrink F., Gordon A.J. (2020). Associations between stopping prescriptions for opioids, length of opioid treatment, and overdose or suicide deaths in US veterans: Observational evaluation. BMJ.

[34] Decaroli M.C., Rochira V. (2016). Aging and sex hormones in males. Virulence.

[35] De Vries F., Bruin M., Lobatto D.J., Dekkers O.M., Schoones J.W., Van Furth W.R., Pereira A.M., Karavitaki N., Biermasz N.R., Najafabadi A.H.Z. (2019). Opioids and Their Endocrine Effects: A Systematic Review and Meta-analysis. J. Clin. Endocrinol. Metab..

[36] Davis S.R., Baber R., Panay N., Bitzer J., Perez S.C., Islam R.M., Kaunitz A.M., Kingsberg S.A., Lambrinoudaki I., Liu J. (2019). Global Consensus Position Statement on the Use of Testosterone Therapy for Women. J. Clin. Endocrinol. Metab..

